# Incidence and Location of Osteochondral Lesions of the Talus Associated With Ankle Fractures Evaluated by Magnetic Resonance Imaging

**DOI:** 10.7759/cureus.94330

**Published:** 2025-10-11

**Authors:** Futoshi Morio, Shota Morimoto, Shigeo Fukunishi, Yoshinobu Masumoto, Masahiro Komeda, Tomokazu Fukui, Akira Okayama, Tokuhide Moriyama, Toshiya Tachibana, Tomoya Iseki

**Affiliations:** 1 Department of Orthopedic Surgery, Hyogo Medical University, Nishinomiya, JPN; 2 Department of Orthopedic Surgery, Nishinomiya Kaisei Hospital, Nishinomiya, JPN; 3 Department of Orthopedic Surgery, Kawasaki Hospital, Kawasaki, JPN; 4 Department of Orthopedic Surgery, Yusei Hospital, Itami, JPN; 5 Department of Orthopedic Surgery, Osaka Kaisei Hospital, Osaka, JPN; 6 Department of Orthopedic Surgery, JCHO Kobe Central Hospital, Kobe, JPN; 7 Department of Orthopedic Surgery, Takarazuka City Hospital, Takarazuka, JPN

**Keywords:** ankle fracture, cross-sectional study, magnetic resonance imaging, osteochondral lesion of the talus, residual symptoms

## Abstract

Objective: Osteochondral lesions of the talus (OLTs) are known to be one of the causes of residual pain after ankle fractures. Few reports investigate the incidence and location of OLTs associated with ankle fractures using magnetic resonance imaging (MRI) at the time of injury. The purpose of this study is to determine the incidence and anatomical distribution of OLTs on acute MRI in patients with ankle fractures using MRI at the time of injury.

Methods: Consecutive 84 patients with ankle fractures who underwent MRI within one week of injury between October 2021 and May 2023 were included in this study. The diagnosis of an ankle fracture and evaluation of the fracture type were performed with plain radiographs and computed tomography, and an MRI was performed to assess the incidence and location of OLTs. In addition, the incidence of isolated fractures was compared with that of bimalleolar and trimalleolar fractures. After exclusion, the remaining 67 patients were analyzed.

Results: OLTs were found in 18 of 67 patients (26.9%). All lesions were isolated talar lesions; the posterolateral aspect of the talus occurred most frequently in 7 of 18 patients (38.9%). In addition, OLTs occurred in 12 of 29 patients (41.4%) with isolated malleolar fractures, which was significantly higher than the combined incidence of bimalleolar and trimalleolar cases (6/38: 15.8%) (*P=0.027*).

Conclusion: Acute MRI detects OLTs in over one quarter of ankle fractures, most commonly in the posterolateral talus. Additionally, the incidence of OLTs was significantly higher in patients with isolated malleolar fractures.

## Introduction

Ankle fractures are one of the most common lower extremity fractures, accounting for approximately 9% of all fractures [1]. Despite receiving appropriate treatment, up to 50% of patients with ankle fractures report residual symptoms during both short- and long-term follow-up [2]. One of the most frequently reported complaints is persistent pain, which can significantly impair patients' daily functioning [1,2]. A contributing factor to this residual pain is osteochondral lesions of the talus (OLTs), which may arise either from the initial trauma associated with the ankle fracture or from chronic cartilage damage caused by residual ankle mortise instability [3,4].

According to several previous studies, the reported incidence of OLTs associated with ankle fractures varies widely, ranging from approximately 10% to 70% [5-7]. Regarding the anatomical distribution of these lesions, some reports indicate that the posterolateral aspect of the talus is the most commonly affected site, while others report a predominance in the anteromedial aspect [8-10]. The variability in the reported incidence and location of OLTs may be attributed to differences in diagnostic imaging modalities, such as plain radiography, computed tomography (CT), magnetic resonance imaging (MRI), or arthroscopy, as well as variations in the timing of assessment following ankle fractures.

MRI is an important imaging modality used to evaluate articular cartilage, subchondral bone alterations, and soft-tissue injuries; it is considered the gold standard for assessing chondral and osteochondral lesions of the ankle joint [11]. Although several previous studies have evaluated OLTs associated with ankle fractures using MRI [4,6,8,12,13], only two have assessed OLTs at the time of ankle fracture [4,6]. However, both studies included only ankle fracture patients who received surgical treatment. Additionally, these studies did not investigate the location of OLTs. To our knowledge, no reports have investigated the incidence and location of OLTs associated with ankle fractures using MRI at the time of injury in patients who received both conservative and surgical treatment.

There have been several reports on the incidence of OLTs associated with ankle fractures and their relationship to fracture type [4,7,8]. Nosewicz et al. reported that OLTs occurred in all Lauge-Hansen stage III or IV ankle fractures, but they reported no significant association between OLTs and ankle fracture type [7]. Ozcan et al. also found no significant association between the type of ankle fracture and the presence of OLTs [8]. As aforementioned, the relationship between OLT incidence and fracture type remains controversial.

The primary aim of this study is to clarify the incidence and location of OLTs associated with ankle fractures using MRI at the time of injury. The secondary aim was to compare the incidence of OLTs among fracture types. We hypothesized that patients with more complex fracture patterns (bimalleolar and trimalleolar) would demonstrate higher OLT incidence than those with isolated malleolar fractures.

This article was previously presented as a meeting abstract at the Japanese Orthopaedic Association Annual Meeting on May 25, 2024.

## Materials and methods

A multicenter cross-sectional study was conducted at seven hospitals between October 2021 and May 2023. Institutional review board approval (No. 3894) was obtained prior to the initiation of this project. This study was conducted in accordance with the Declaration of Helsinki, and informed consent was obtained from all patients.

Consecutive 84 patients with ankle fractures who were able to undergo MRI within one week of injury between October 2021 and May 2023 were included in this study. Exclusions were patients younger than 18 years or older than 70 years and patients with a previous history of surgery on the affected lower extremity, systemic inflammatory disease, chronic lateral ankle instability (CLAI), OLTs, and osteoarthritis.

The radiographic evaluation was reviewed using an image storage and communication system (PACS; FujiFilm Medical Synapse Version 5.5.000 V4.1, Tokyo, Japan). The diagnosis of ankle fracture in all patients was performed based on plain ankle radiographs and CT. All subjects underwent an MRI of the affected ankle on either a 1.5T scanner or a 3.0T scanner within one week of injury. All MRI examinations included coronal and sagittal T1- and T2-weighted images and coronal, sagittal, and axial T1-weighted short-tau inversion recovery (STIR) images. Images were taken in the supine position, with the affected ankle in a neutral position. OLT evaluation was performed by an orthopedic surgeon (F.M.) with experience evaluating musculoskeletal imaging.

OLTs were diagnosed based on characteristic findings, including hypointense areas on T1-weighted images and signal rim changes on T2-weighted images, as previously described [14]. All OLTs were classified according to the classification system reported by Hepple et al. as follows: Stage I, articular cartilage damage only; Stage IIA, cartilage injury with underlying fracture and surrounding bony edema; Stage IIB, no bony edema; Stage III, detached, but no displaced fragment; Stage IV, displaced fragment; Stage V, subchondral cyst formation (Table 1) [15].

**Table 1:** Classification of Osteochondral Lesion Abbreviation: BME, bone marrow edema. Table created by Futoshi Morio, based on Hepple et al. [15].

Stage	Description
Ⅰ	Articular cartilage damage only
Ⅱa	Cartilage injury with underlying fracture and surrounding bony edema
Ⅱb	Without bony edema
Ⅲ	Detached, but undisplaced fragment
Ⅳ	Displaced fragment
Ⅴ	Subchondral cyst formation

The OLT's location was evaluated by dividing the talar dome into nine regions.

During the course of this study, it was hypothesized that the incidence of OLTs may vary depending on the type of ankle fracture. Therefore, patients were divided into three groups depending on fracture type (isolated malleolar, bimalleolar, and trimalleolar), and the incidence for each was then evaluated. In addition, the incidence of patients with isolated malleolar fractures was compared to that of patients with bimalleolar and trimalleolar fractures.

Statistical analysis was performed using IBM SPSS Statistics for Windows, Version 19 (Released 2010; IBM Corp., Armonk, New York). The assumption of normality for the radiographic parameters was evaluated using the Shapiro-Wilk test. The comparison of means was performed using the Student's t-test or the Mann-Whitney U test. A comparison of frequencies was performed using the chi-square test. P-values less than .05 were considered statistically significant.

## Results

Among the initially enrolled 84 patients, 10 patients who were younger than 18 years or older than 70 years, two patients with a previous history of systemic inflammatory disease, one patient with a previous history of CLAI, and four patients with a previous history of osteoarthritis were excluded from the study. The remaining 67 patients were analyzed. The 67 patients comprised 33 males (49.3%) and 34 females (50.7%) with a mean age at the time of injury of 52.1 years (SD ±15.9). Among the 67 patients, 57 (85.1%) received surgical treatment, and the remaining 10 (14.9%) received conservative treatment. The fracture types were isolated malleolar fractures in 29 cases (43.3%), bimalleolar fractures in 17 cases (25.4%), and trimalleolar fractures in 21 cases (31.3%). A total of 12 patients (17.9%) admitted tobacco use, and 11 patients (16.4%) had a history of diabetes mellitus (Table 2).

**Table 2:** Patient Characteristics of the Study Population (n=67) Abbreviation: BMI, body mass index.

Variable	Value
Gender, M/F	33/34
Age at time of injury, y	52.1±15.9
Side, L/R	36/31
BMI, kg/m²	23.8±3.4
Treatment type	
Operative	57
Conservative	10
Fracture type	
Isolated malleolar	29
Bimalleolar	17
Trimalleolar	21
Tobacco use	12
Diabetes mellitus	11

OLTs were present in 18 of 67 patients (26.9%), all of which were isolated talar lesions. Regarding patient characteristics, there were no significant differences between the OLT and non-OLT groups in terms of gender (P=0.41), age (P=0.52), BMI (P=0.81), side injured (P=0.85), history of diabetes (P=0.24), or smoking (P=0.49). According to Hepple's classification, there were 14 cases of Stage I OLTs (77.8%) and four cases of Stage IIA OLTs (22.2%), with no Stage IIB, III, IV, or V OLTs. OLTs occurred in 12 of 29 (41.4%) isolated cases, 3 of 17 (17.6%) bimalleolar cases, and 3 of 21 (14.3%) trimalleolar cases (Table 3).

**Table 3:** Incidence of Osteochondral Lesion of the Talus (OLT) by Fracture Type

Hepple's Classification	Isolated Malleolar (n=29)	Fracture Types of All Lesions		
		Bimalleolar (n=17)	Trimalleolar (n=21)	Total (n=67)
Stage Ⅰ	10	2	2	14 (77.8%)
Stage Ⅱa	2	1	1	4 (22.2%)
Total	12 (41.4%)	3 (17.6%)	3 (14.3%)	18/67 (26.9%)

In addition, OLTs occurred in 12 of 29 patients (41.4%) with isolated malleolar fractures, which was significantly higher than the combined incidence of bimalleolar and trimalleolar cases (6/38: 15.8%) (P=0.027) (Table 4).

**Table 4:** Cross-Table Between Fracture Type and Presence of Osteochondral Lesion of the Talus (OLT)

Case	OLT (+), n (%)	OLT (-), n (%)	Total
Isolated malleolar	12 (41.4%)	17 (58.6%)	29
Bimalleolar + Trimalleolar	6 (15.8%)	32 (84.2%)	38
Total	18	49	67
			P=0.027

**Figure 1:**
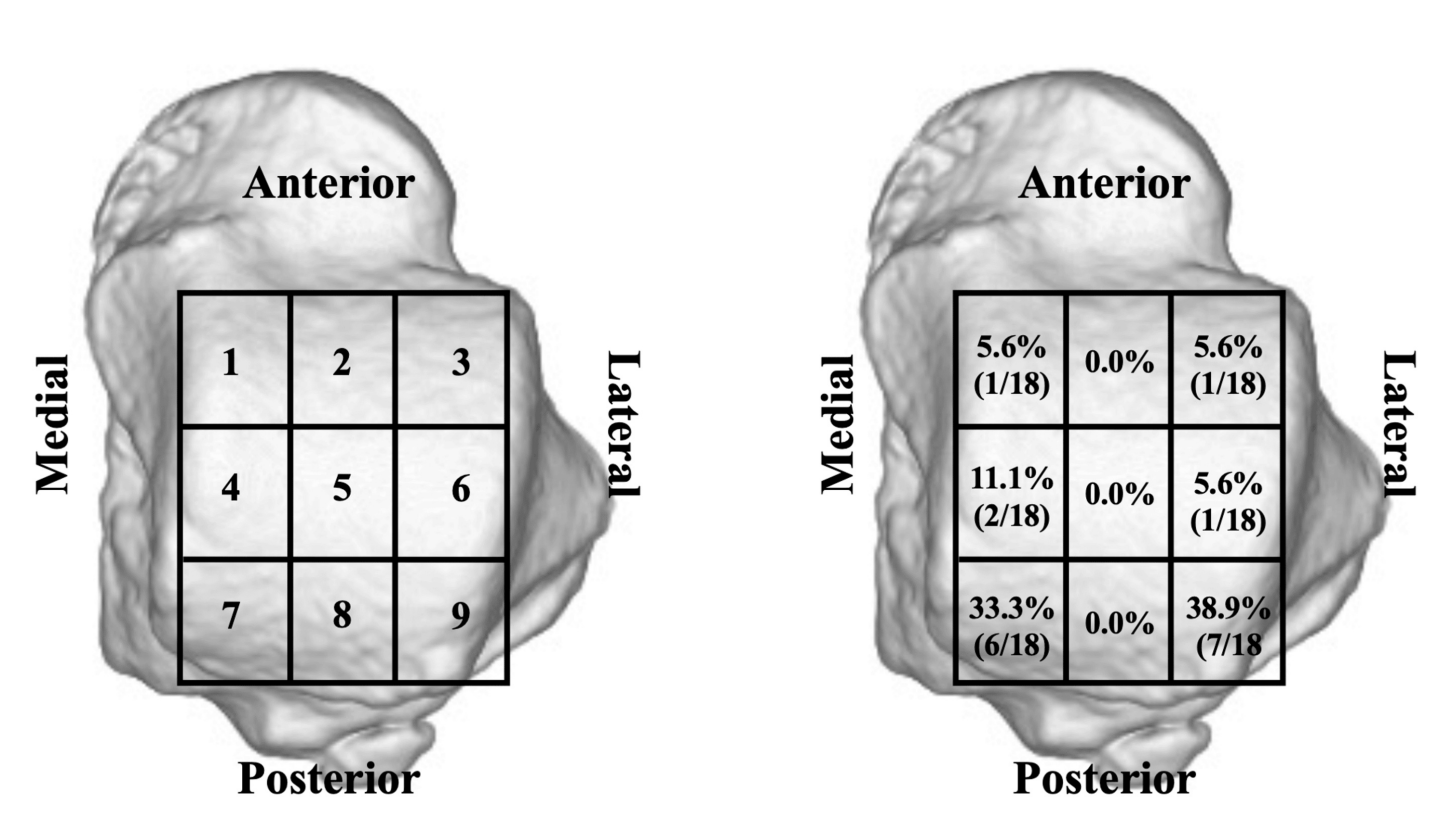
Location of OLTs—nine-grid map of the talar dome This figure is an original image created by the authors.

The most common site of OLT was the posterolateral aspect of the talus in 7 of 18 patients (38.9%), followed by the posteromedial aspect in 6 of 18 cases (33.3%), with approximately 70% of OLTs occurring posterior to the talus (Figure 1).

## Discussion

The present study investigated the incidence and location of OLTs associated with ankle fractures, as evaluated by MRI at the time of injury. The principal findings of this study show a 26.9% incidence rate of OLTs occurring at the time of ankle fracture. The most common location of OLT was the posterolateral aspect of the talus (38.9%), followed by the posteromedial aspect (33.3%), with approximately 70% of OLTs occurring posterior to the talus. In addition, the incidence of OLT was significantly higher in isolated malleolar fracture cases (12 of 29 patients: 41.4%) than the combined incidence of bimalleolar and trimalleolar cases (6 of 38 patients: 15.8%).

In cases of ankle fractures, up to 50% of patients complain of residual symptoms, especially pain [2]. OLTs are a defect of the cartilage and subchondral bone of the talar dome and are reported to be one of the main causes of residual pain after an ankle fracture. The etiology of OLTs associated with ankle fractures is generally categorized as either acute or chronic. In acute cases, OLTs are thought to result from direct impact between the talus and the tibia during an ankle sprain at the time of fracture. In contrast, chronic OLTs are believed to arise from repetitive microtrauma secondary to persistent ankle instability. Previous reports have indicated that the incidence of OLTs associated with ankle fractures is approximately 10-70%, with some variation across reports [5-7]. Possible reasons for variation in the incident rate across reports include OLTs being evaluated with different diagnostic imaging modalities and at different times following an ankle fracture.

The initial imaging modality for evaluating OLTs is typically plain radiography. However, in cases of acute injury with non-displaced lesions, plain radiographs may fail to reveal any abnormal findings [16-19]. Loomer et al. have reported a 50% prevalence of OLTs on plain radiographs [18], indicating that plain radiographs are less sensitive in assessing articular cartilage surface integrity [20]. A CT following an ankle fracture is often used to visualize fracture characteristics in the preoperative workup. Nosewicz et al. used postoperative CT to investigate OLTs associated with ankle fractures and reported an incidence of 10% [7]. Lambers et al. also reported the incidence of OLTs associated with ankle fractures with syndesmosis injuries using CT within approximately one month after surgery and determined the incidence to be 14% [21]. Togher et al. examined OLTs associated with ankle fractures using CT at the time of injury and reported an incidence rate of 50.9% [9]. However, while CT is useful for evaluating subchondral bone, it is not useful for evaluating chondral lesions [8]. Although Hintermann et al. studied OLTs associated with ankle fractures using arthroscopy and reported an incidence of 69.4% [5], arthroscopy has been reported to be less sensitive for deep lesions [22-24].

MRI is considered the gold standard for evaluating OLTs, as it offers superior sensitivity for detecting cartilage damage, subchondral bone changes, and soft-tissue injuries compared with other imaging modalities [11,25,26]. Several studies have investigated the incidence of OLTs associated with ankle fractures using MRI [4,6,8,12,13]. Among them, only two have evaluated OLTs at the time of fracture [4,6]. Takao et al. prospectively studied 92 ankle fractures using MRI and arthroscopy and reported an OLT incidence rate of 70.7% [4]. Boraiah et al. also retrospectively investigated 153 ankle fractures and reported that OLTs occurred in 17% of cases [6]. However, both studies only analyzed ankle fractures that were treated surgically. In this study, the incidence of OLTs was evaluated using MRI at the time of ankle fracture, included patients treated with either conservative or operative treatment, and found the incidence to be 26.9% (18 of 67 patients). Furthermore, the present study compared the incidence of OLTs across fracture types and found that it was significantly higher in isolated malleolar fractures (12 of 29 patients: 41.4%) than in bimalleolar and trimalleolar fractures combined (6 of 38 patients: 15.8%). Park et al. reported the relationship between radiographic ankle instability and OLTs following ankle inversion injuries [27]. In this study, the incidence of OLTs decreased with increased tibiotalar tilt angle due to deltoid or syndesmosis injury, and the presence of OLTs was associated with a decreased tibiotalar tilt angle. They reported that the greater degree of bony containment of the ankle mortise surrounding the talus could considerably prevent mechanical instability of the ankle joint following ankle injury; however, such a structure would increase the likelihood of bony collision and subsequent OLTs following ankle injury. It was hypothesized that injuries to the deltoid ligament or syndesmosis are relatively rare in isolated malleolar fractures, and that the greater bony containment of the ankle mortise surrounding the talus may result in a lower incidence of OLTs.

Several reports have investigated the location of OLTs associated with ankle fractures. Togher et al. used CT at the time of injury to investigate the location of OLTs and demonstrated that the posterolateral aspect of the talus was the most common, with more than 50% of OLTs occurring posterior to the talus [9]. Kraniotis et al. and Nosewicz et al. used postoperative CT to examine the location of OLTs and both reported that OLTs were more common in the posterolateral aspect of the talus [7,10]. On the other hand, Ozcan et al. examined the occurrence of OLTS using MRI two months after injury and reported that the most common site was the anteromedial aspect of the talus [8]. In the present study, OLTs occurred most frequently on the posterolateral aspect of the talus (38.9%), followed by the posteromedial aspect (33.3%), with approximately 70% occurring in the posterior part of the talus, similar to results from several previous reports. It has been reported that most ankle joint dislocation fractures are associated with posterior dislocation [28]. On the other hand, posterior dislocation is also reported to be common in cases of ankle sprain dislocation without fracture [29]. Therefore, the ankle joint is more likely to be stressed posteriorly at the time of trauma, and the posterior talus and tibia are more likely to impinge on each other. Therefore, in this study, we considered that the OLTs associated with ankle fractures are more likely to occur posteriorly.

This study has several limitations. First, the sample size was small. Second, because this is a multicenter study, the MRIs are not identical. Either a 1.5T or 3.0T scanner system was used. It has been reported that no comparison exists in the literature regarding the diagnostic accuracy of 1.5 or 3.0 T scanners in the evaluation of a chondral lesion [6]. Third, since the evaluation and detection of OLT were performed by a single orthopedic surgeon, an assessment of observer bias could not be assessed. Fourth, MRI may overdiagnose the OLTs due to edema from the fracture. Cartilage and subchondral damages can be seen using MRI, but it has been reported that subchondral edema is often exaggerated and can be mistaken for chondral injury [30]. However, because evaluation of CT images cannot account for the presence of isolated, pure chondral injuries, in this study, this study used MRI to investigate the incidence and location of OLTs that occur at the time of injury. Moreover, it cannot be denied that the OLTs existed prior to the time of the ankle fracture injury. However, although the present study excluded cases with a history of CLAI, it may not have been able to exclude congenital OLTs. Finally, no evaluation of the deltoid ligament or syndesmosis was performed, and instability on imaging was not assessed.

## Conclusions

In conclusion, the incidence of OLTs occurring at the time of ankle fracture injury was 26.9%. Additionally, the incidence of OLTs was significantly higher in patients with an isolated malleolar fracture. OLTs associated with ankle fractures at the time of injury occurred more frequently in the posterolateral aspect and the posteromedial aspect of the talus. Although the clinical outcome of OLTs associated with ankle fractures at the time of injury requires further investigation, orthopedic surgeons should keep in mind that OLTs are a complication resulting from ankle fractures when proceeding with treatment.
